# Supplementary material for: Maternal skepticism regarding children’s vaccination in Jordan: Investigating the knowledge, attitude, and adherence
Source: PLoS One. 2024 Jul 10;19(7):e0305209. doi: 10.1371/journal.pone.0305209 (PMC11236176; doi:10.1371/journal.pone.0305209)
Supplement: Attachment — Submitted filename: PONE-D-24-05111_reviewerNote_RD_Maternal Skepticism.docx [file pone.0305209.s001.docx]

PLOS ONE Maternal Skepticism Regarding Children's Vaccination in Jordan: Investigating the Knowledge, Attitude, and Adherence

Manuscript Number: PONE-D-24-05111

**Introduction and Literature review**

- A clear and concise overview of vaccination programs and their importance to healthcare systems is provided in the Introduction.
- It was intended to assess parents' knowledge, attitude, and adherence to vaccination and their vaccination choices, however, the literature lacks information from previous studies.
- This section highlights the lack of research on mothers' knowledge, attitudes, and adherence to vaccination in Jordan. To add context to the study's significance, it would be helpful to briefly mention any existing studies or literature gaps that the current research intends to address.
- There is a brief mention of the COVID-19 pandemic's impact on vaccination coverage, which is relevant to the study's findings. A more comprehensive understanding of the research context would be provided by focusing on the specific challenges faced by Jordan during the pandemic and their effects on vaccination programs.
- It would be helpful to include more context about the socioeconomic, cultural, and political factors that influence vaccination practices in Jordan in the introduction.
- The literature review section of the report should be organized logically and coherently to improve the flow of ideas. This is a suggested flow that might be helpful.
- The report should begin by providing an overview of the vaccination program and highlighting its health and economic benefits.
- Then, discuss a variety of studies that have shown the cost-effectiveness of vaccination programs, in particular the WHO report, which states that vaccine-preventable diseases have resulted in a significant reduction in child mortality.
- Describe the impact of successful vaccination campaigns on eradicating or reducing disease.
- Describe UNICEF-Jordan's collaboration with the Jordanian Ministry of Health to implement vaccination programs.
- Give an overview of Jordan's free child vaccination program, including vaccines provided and vaccination rates.  It is also important to identify the elective vaccinations.
- Describe fluctuations in vaccination coverage and disease prevalence in Jordan and the Middle East.
- The skepticism and hesitation of mothers to receive vaccinations, as identified in previous studies, needs to be addressed.
- Discuss the impact of the COVID-19 pandemic on Jordanian vaccination coverage.
- Stress the critical importance of parental (especially maternal) cooperation in ensuring a child's successful vaccination.
- Find out what factors are influencing vaccination decisions and why some parents’ hesitancy to vaccinate.

Using this flow of ideas, the literature review section of the report will present a comprehensive overview of vaccination programs, their impact, challenges faced, and Jordan's specific context. Moreover, it will demonstrate the importance of the proposed study in addressing existing research gaps.

Overall, the Introduction effectively sets the stage for the study by providing relevant background information on vaccination programs in Jordan and identifying key research gaps to be addressed. Some minor revisions, such as citation consistency and further elaboration on specific topics, would enhance the clarity and comprehensiveness of the section.

**Materials and methods**

- In the section, convenience sampling is mentioned briefly, but recruitment and selection of participants aren't described.
- It would be helpful to provide more detail about the specific added questions and their relevance to the study objectives, especially as they relate to the role of pharmacists in the study.
- There is a clear definition of the eligibility criteria for participants. However, a brief explanation of why mothers with children under 12 are included would be beneficial.
- This section explains how the questionnaire was developed, including how it was adapted from previous literature and how linguistic and content authenticity was ensured.
- Demographic variables are comprehensive and relevant to the study objectives.
- Using Likert scales and multiple-choice items to assess attitudes, knowledge, and adherence enhances the questionnaire's comprehensiveness.
- The scoring system for each section of the questionnaire is clearly explained.
- The inclusion of items that assess maternal attitudes and adherence to elective vaccinations is a valuable addition to the questionnaire.
- Validity of the questionnaire was sought efficiently
- Informed consent procedures, IRB approval, and confidentiality measures are clearly outlined during the study.
- The informed consent process, including a letter of informed consent, is described thoroughly and demonstrates a commitment to ethical research practices.

**Results**

- A detailed summary of the findings of Table 1, Table 2, Table 3, Table 4, and Table 5 is provided in the results section. For simplicity, the results can be summarized by referring to the following table without repeating the same information in the text. The highest and lowest ranked items do not need to be interpreted at this point and can be discussed later
- The inclusion of multi-response questions about reasons for non-participation in elective vaccinations as well as sources of information enhances the analysis
- It is essential to ensure consistency in terminology and language throughout the entire section. In order to enhance clarity, either "mothers" or "participants" should be consistently used to refer to the study sample.
- The tables stand on their own, but they are redundant with those presented in the text, so it is suggested to omit the repeated information in the text and keep it in the table. Likewise, the table format should be adjusted academically and according to the journal's requirements
- The mean scores of mothers' knowledge are given as 17.8 (SD = 3.97) in the text but are given differently in table 1.

**Discussion**

- This section effectively integrates findings from previous studies to contextualize the current study's findings. It would be helpful if a brief summary of the key findings of these studies could be provided, along with how those findings relate to the findings of the current study.
- It presents the findings in a logical sequence and is well-structured and coherent.
- It effectively acknowledges both the positive aspects of mother's knowledge, attitudes, and adherence to vaccinations, as well as the challenges, such as modest awareness of elective vaccines and concerns about costs.
- The discussion provides valuable insights into similarities and differences across different contexts by comparing the study's findings with those of previous research. It would be useful to discuss potential reasons for discrepancies, such as cultural or healthcare system differences, to enhance the depth of the analysis."
- The discussion concludes with practical recommendations to address the identified challenges. Healthcare professionals and policymakers will benefit from these recommendations, which are aligned with the study's objectives and findings.
- The language used is clear and professional, contributing to the overall readability.
- There are some studies referred to in the discussion that should be included in the literature review section of the report while others should be moved completely earlier in the report.
- As a limitation, it is better to mention that the sample did not represent Jordanian populations since it was based mainly on Jordan's largest cities and excluded the southern and rural areas. This can also be enhanced by offering suggestions for future researchers to overcome this problem
- There was an effective identification of barriers to mothers' adherence to elective vaccinations in the discussion.
