# Supplementary material for: Maternal skepticism regarding children’s vaccination in Jordan: Investigating the knowledge, attitude, and adherence
Source: PLoS One. 2024 Jul 10;19(7):e0305209. doi: 10.1371/journal.pone.0305209 (PMC11236176; doi:10.1371/journal.pone.0305209)
Supplement: Attachment — Submitted filename: Maternal Skepticism Regarding Children.docx [file pone.0305209.s002.docx]

**Maternal Skepticism Regarding Children's Vaccination in Jordan: Investigating the Knowledge, Attitude, and Adherence**

Review report

Dear authors,

Thank you for giving me the chance to review your high standard research, however I have some comments to raise:

**Abstract:**

Clear but mention the vaccination rate in Jordan as a background information, and those elective vaccination you consider in your study.

**Introduction:**

You mention a high vaccination rate, and only 5% of children were not vaccinated, can you mention more regarding the those 5% in Jordan, are they from the refugees or Jordanian kindly clarify…, what about the vaccination coverage among refugees in Jordan. Or other marginalized group, as in Jordan even the Bedouin are receiving a high coverage of vaccination through mobile primary health clinics.

You mention one paragraph : Despite the children's vaccination programs in place…” concerning the importance of vaccination and here you mention the routine vaccination programs carried in many other countries, however the vaccination coverage in Jordan is very high…what is the significant of your study, what are the elective vaccination that you are studying or looking for, I feel the introduction is very general try to be specify to what exactly you would like to justify your study . And what your study will add more than the study of Batarseh et al. mentioning the adherence to COVID-19 vaccination was already previously studied, what are other elective vaccination that you try to study here.

Kindly justify the study more and give it more application perspectives in the future.

Aim of the study need to specify what you are looking for.

**Material and method:**

Why do you need a sample size calculation when you conducted an online study and a convenient sample. kindly explain in the statistical analysis and justify your sample size.

Study instrument: what is the validity and reliability of the original questionnaire and applied to which culture or population, and what is the Cronbach alpha for the translated questionnaire. Following the translation have you adapted any of the questions?

The last item of the adherence section was “I give my child elective vaccines (which are not included in the national vaccination program)”. I believe this is very general question??? And probably wound need an open ended question since multiple choice answers for the reasons might be leading answers and might confound the results.

“The initial query sought the respondent's confirmation that their age surpassed the threshold of 18 years and that they had at least one child at the age of 12 years or less” how do you control this in an online questionnaire, kindly explain.

“After completing the questionnaire, the participants were given the option to provide an email address to which they could receive information regarding children’s vaccinations” explain the purpose of that

**Results**

Table 2: “Are you aware of elective vaccines available in the private sector and not included in the national vaccination program?” this is not specific question, when you never mentioned those elective vaccines that you consider in your study.

Table 5 report the reasons for not adhering to elective vaccination, not knowing the vaccine and not knowing where to get it…related to the same concept since elective vaccines was not mentioned earlier in the study and not introducing the mothers for the elective vaccines etc. sending the parents of the MOH information guild means that there is a policy measures in place to clarify ambiguity related o vaccination….what this study would add. And my self I find this result is very logical…..and expected.

Table 3: Pharmacies should provide and administer vaccines. I don’t understand the purpose of this question? And the relation to the attitudes when the vaccination are provided free of cost the PHC centers? Some how I got a feeling that those who selected strongly disagree are the same participants …since the numbers were close to each other…can you check if those participants might miss understood the scale, and there is some confounding factor.

Any correlational results with mother’s background. And demographic information?

**Discussion:**

papers (28)…one study, where?

“Pharmacists in developed countries are considered approved and reliable vaccination providers which boosts vaccination coverage and healthcare financial savings (31, 32, 33). Therefore, it is important to promote pharmacists’ role as vaccination providers to spread awareness and a more positive attitude towards vaccination-related advantages. This is not included in your research purpose.

Discussion needs to focus on the results as well as the applicability of the study.

**Over all review:**

English, well written. The study needs further analysis to assess the related factors, clearness regarding the elective vaccination was not found in the study making the study results very predictable.

Thank you,
