# Supplementary material for: Maternal skepticism regarding children’s vaccination in Jordan: Investigating the knowledge, attitude, and adherence
Source: PLoS One. 2024 Jul 10;19(7):e0305209. doi: 10.1371/journal.pone.0305209 (PMC11236176; doi:10.1371/journal.pone.0305209)
Supplement: Attachment — Submitted filename: Responses to reviewers comments_2.docx [file pone.0305209.s003.docx]

Dear respected editor and reviewers,

We would like to thank the editor and reviewers for the time and effort invested in reviewing our manuscript titled “Maternal Skepticism Regarding Children's Vaccination in Jordan: Investigating the Knowledge, Attitude, and Adherence”.

We appreciate the thoughtful and constructive feedback and carefully considered each comment and suggestion in preparing this revised manuscript. As you will see in the below responses to the reviewers' comments, we have made the requested revisions to the manuscript to address the concerns raised.

We hope that the revised manuscript meets the standards of your esteemed journal, and we look forward to your feedback. Once again, we are grateful for the opportunity to resubmit my work to your journal. Please note that all the changes made to the manuscript are in yellow highlight to facilitate the review process. We have also included a point-by-point response to each of the comments made by the reviewer.

| **Reviewer’s Comments to the Author** | **Responses** |
| --- | --- |
| **GENERAL COMMENTS:** | Thank you very much for your positive comments! The revisions are made as described below. |
| **Reviewer 1** | |
| **REQUESTED REVISIONS:** |  |
| **Introduction and Literature review**  - A clear and concise overview of vaccination programs and their importance to healthcare systems is provided in the Introduction. | Thank you. We appreciate your feedback! |
| - It was intended to assess parents' knowledge, attitude, and adherence to vaccination and their vaccination choices, however, the literature lacks information from previous studies.  - This section highlights the lack of research on mothers' knowledge, attitudes, and adherence to vaccination in Jordan. To add context to the study's significance, it would be helpful to briefly mention any existing studies or literature gaps that the current research intends to address. | The literature is updated to address the comments.  A section was dedicated to address the Jordanian context. |
| - There is a brief mention of the COVID-19 pandemic's impact on vaccination coverage, which is relevant to the study's findings. A more comprehensive understanding of the research context would be provided by focusing on the specific challenges faced by Jordan during the pandemic and their effects on vaccination programs. | A section on COVID-19 impact was highlighted. |
| - It would be helpful to include more context about the socioeconomic, cultural, and political factors that influence vaccination practices in Jordan in the introduction. | Thank you! A section was dedicated to the Jordanian context to address this comment. |
| - The literature review section of the report should be organized logically and coherently to improve the flow of ideas. This is a suggested flow that might be helpful.  • The report should begin by providing an overview of the vaccination program and highlighting its health and economic benefits.  • Then, discuss a variety of studies that have shown the cost-effectiveness of vaccination programs, in particular the WHO report, which states that vaccine-preventable diseases have resulted in a significant reduction in child mortality.  • Describe the impact of successful vaccination campaigns on eradicating or reducing disease.  • Describe UNICEF-Jordan's collaboration with the Jordanian Ministry of Health to implement vaccination programs.  • Give an overview of Jordan's free child vaccination program, including vaccines provided and vaccination rates. It is also important to identify the elective vaccinations.  • Describe fluctuations in vaccination coverage and disease prevalence in Jordan and the Middle East.  • The skepticism and hesitation of mothers to receive vaccinations, as identified in previous studies, needs to be addressed.  • Discuss the impact of the COVID-19 pandemic on Jordanian vaccination coverage.  • Stress the critical importance of parental (especially maternal) cooperation in ensuring a child's successful vaccination.  • Find out what factors are influencing vaccination decisions and why some parents’ hesitancy to vaccinate.  Using this flow of ideas, the literature review section of the report will present a comprehensive overview of vaccination programs, their impact, challenges faced, and Jordan's specific context. Moreover, it will demonstrate the importance of the proposed study in addressing existing research gaps. | The manuscript (Introduction and Background Section) is reorganized according to your suggestion into subheadings. |
| Overall, the Introduction effectively sets the stage for the study by providing relevant background information on vaccination programs in Jordan and identifying key research gaps to be addressed. Some minor revisions, such as citation consistency and further elaboration on specific topics, would enhance the clarity and comprehensiveness of the section. | Thank you! The introduction including the aims section is updated. |
| **Materials and methods**  - In the section, convenience sampling is mentioned briefly, but recruitment and selection of participants aren't described. | The participation was restricted to those who meet the inclusion criteria as described in the sampling and setting section, Further details are discussed in the data collection section. |
| - It would be helpful to provide more detail about the specific added questions and their relevance to the study objectives, especially as they relate to the role of pharmacists in the study. | A panel of experts suggested that the research will add valuable insights into the pharmacists’ roles in vaccination as the literature lacks this piece. More specifically, elective vaccines are primarily available in pharmacies, therefore, it is important to assess mothers' perspectives of the pharmacists’ role in vaccination. |
| - There is a clear definition of the eligibility criteria for participants. However, a brief explanation of why mothers with children under 12 are included would be beneficial. | As children reach the age of 12 years, the vaccination recommendations by the Centers for Disease Control and Prevention (CDC) change drastically into the *preteen* vaccination recommendation which is not the focus of this study.  <https://www.cdc.gov/vaccines/parents/by-age/years-11-12.html> |
| • This section explains how the questionnaire was developed, including how it was adapted from previous literature and how linguistic and content authenticity was ensured.  • Demographic variables are comprehensive and relevant to the study objectives.  • Using Likert scales and multiple-choice items to assess attitudes, knowledge, and adherence enhances the questionnaire's comprehensiveness.  • The scoring system for each section of the questionnaire is clearly explained.  • The inclusion of items that assess maternal attitudes and adherence to elective vaccinations is a valuable addition to the questionnaire.  • Validity of the questionnaire was sought efficiently  • Informed consent procedures, IRB approval, and confidentiality measures are clearly outlined during the study.  • The informed consent process, including a letter of informed consent, is described thoroughly and demonstrates a commitment to ethical research practices. | Thank you very much! |
| **Results**  • A detailed summary of the findings of Table 1, Table 2, Table 3, Table 4, and Table 5 is provided in the results section. For simplicity, the results can be summarized by referring to the following table without repeating the same information in the text. The highest and lowest ranked items do not need to be interpreted at this point and can be discussed later. | The results section is modified by deleting the narratives on the highest and lowest ranked responses. |
| • The inclusion of multi-response questions about reasons for non-participation in elective vaccinations as well as sources of information enhances the analysis | Thank you! |
| • It is essential to ensure consistency in terminology and language throughout the entire section. In order to enhance clarity, either "mothers" or "participants" should be consistently used to refer to the study sample. | Done. |
| • The tables stand on their own, but they are redundant with those presented in the text, so it is suggested to omit the repeated information in the text and keep it in the table. Likewise, the table format should be adjusted academically and according to the journal's requirements | Done! |
| • The mean scores of mothers' knowledge are given as 17.8 (SD = 3.97) in the text but are given differently in table 1. | Thanks for your note. The error was corrected. |
| **Discussion**  • This section effectively integrates findings from previous studies to contextualize the current study's findings. It would be helpful if a brief summary of the key findings of these studies could be provided, along with how those findings relate to the findings of the current study. | Thank you!  Updated. |
| • It presents the findings in a logical sequence and is well-structured and coherent.  • It effectively acknowledges both the positive aspects of mother's knowledge, attitudes, and adherence to vaccinations, as well as the challenges, such as modest awareness of elective vaccines and concerns about costs. | Thank you! |
| • The discussion provides valuable insights into similarities and differences across different contexts by comparing the study's findings with those of previous research. It would be useful to discuss potential reasons for discrepancies, such as cultural or healthcare system differences, to enhance the depth of the analysis." | Thanks for the valuable comment.  Done! |
| • The discussion concludes with practical recommendations to address the identified challenges. Healthcare professionals and policymakers will benefit from these recommendations, which are aligned with the study's objectives and findings.  • The language used is clear and professional, contributing to the overall readability. | Thank you! |
| • There are some studies referred to in the discussion that should be included in the literature review section of the report while others should be moved completely earlier in the report. | Literature was added to the introduction and background section. |
| • As a limitation, it is better to mention that the sample did not represent Jordanian populations since it was based mainly on Jordan's largest cities and excluded the southern and rural areas. This can also be enhanced by offering suggestions for future researchers to overcome this problem. | Thank you! Added to the limitations section. |
| • There was an effective identification of barriers to mothers' adherence to elective vaccinations in the discussion. | Thank you! |
| **Reviewer 2** | |
| Dear authors,  Thank you for giving me the chance to review your high standard research, however I have some comments to raise: | Thank you! |
| **Abstract**:  Clear but mention the vaccination rate in Jordan as a background information, and those elective vaccination you consider in your study. | Done!  The vaccination rate was not added to the abstract, and deleted from the introduction section as there are no updated references on the current vaccination rate in Jordan. |
| Introduction:  You mention a high vaccination rate, and only 5% of children were not vaccinated, can you mention more regarding the those 5% in Jordan, are they from the refugees or Jordanian kindly clarify…, what about the vaccination coverage among refugees in Jordan. Or other marginalized group, as in Jordan even the Bedouin are receiving a high coverage of vaccination through mobile primary health clinics. | The article did not include information regarding those 5%, and regarding the vaccination coverage among refugees, the literature lacks enough information. introduction is updated to explain this point. |
| You mention one paragraph : Despite the children's vaccination programs in place…” concerning the importance of vaccination and here you mention the routine vaccination programs carried in many other countries, however the vaccination coverage in Jordan is very high…what is the significant of your study, what are the elective vaccination that you are studying or looking for, I feel the introduction is very general try to be specify to what exactly you would like to justify your study . And what your study will add more than the study of Batarseh et al. mentioning the adherence to COVID-19 vaccination was already previously studied, what are other elective vaccination that you try to study here. Kindly justify the study more and give it more application perspectives in the future. | Thanks for the valuable comments, literature has been updated to meet the required recommendations. |
| Aim of the study need to specify what you are looking for. |  |
| Material and method:  Why do you need a sample size calculation when you conducted an online study and a convenient sample. kindly explain in the statistical analysis and justify your sample size. | The calculation was done to ensure the statistical sufficiency of the sample and increase the rigor of the study. |
| Study instrument: what is the validity and reliability of the original questionnaire and applied to which culture or population, and what is the Cronbach alpha for the translated questionnaire. Following the translation have you adapted any of the questions? | The questionnaire was specifically designed for this study based on previous literature. The Cronbach alpha for this study is 0.77. |
| The last item of the adherence section was “I give my child elective vaccines (which are not included in the national vaccination program)”. I believe this is very general question??? And probably wound need an open ended question since multiple choice answers for the reasons might be leading answers and might confound the results. | This is the first study to explore elective vaccination. Therefore, a general question was asked. Future studies are recommended to further discuss this section in specific.  We appreciate your comment and will, for sure, consider it in future studies. |
| “The initial query sought the respondent's confirmation that their age surpassed the threshold of 18 years and that they had at least one child at the age of 12 years or less” how do you control this in an online questionnaire, kindly explain. | The questionnaire was structurally designed with a pair of obligatory queries to ensure adherence to the prerequisites for engagement in the study. The initial query sought the respondent's confirmation that their age surpassed the threshold of 18 years and that they had at least one child at the age of 12 years or less. The second query focused on the agreement of voluntary approval, underpinned by the option of terminating the response at one's discretion.  If the participants checked both as yes, they were considered eligible for the study. |
| “After completing the questionnaire, the participants were given the option to provide an email address to which they could receive information regarding children’s vaccinations” explain the purpose of that | This was an option just in case the link to the vaccination booklet did not work. |
| Results  Table 2: “Are you aware of elective vaccines available in the private sector and not included in the national vaccination program?” this is not specific question, when you never mentioned those elective vaccines that you consider in your study. | The elective vaccines in Jordan are: Hepatitis A, Varicella, Pneumococcal, Flu, Meningococcal vaccines. This is the first study to explore elective vaccination. Therefore, a general question was asked. Future studies are recommended to further discuss this section in specific. |
| Table 5 report the reasons for not adhering to elective vaccination, not knowing the vaccine and not knowing where to get it…related to the same concept since elective vaccines was not mentioned earlier in the study and not introducing the mothers for the elective vaccines etc. sending the parents of the MOH information guild means that there is a policy measures in place to clarify ambiguity related o vaccination….what this study would add. And my self I find this result is very logical…..and expected. | Although the MOH provided a booklet on the mandatory and elective vaccines, from practical experience, we noted that there is a lack of awareness regarding the availability of these vaccines. Therefore, this study aimed to raise awareness of this topic and encourage future research on this specific area. |
| Table 3: Pharmacies should provide and administer vaccines. I don’t understand the purpose of this question? And the relation to the attitudes when the vaccination are provided free of cost the PHC centers? Some how I got a feeling that those who selected strongly disagree are the same participants …since the numbers were close to each other…can you check if those participants might miss understood the scale, and there is some confounding factor. | The elective vaccines are not free. They are only available for purchase in pharmacies and private clinics.  Since the data collection is finished, it is impossible to clarify responses with participants at this stage. We appreciate your comment and will consider it in future studies. |
| Any correlational results with mother’s background. And demographic information? | The current study is exploratory in nature, not meant to analyze associations with demographic factors. The demographic characteristics were provided to give an overview on the study participants.  We appreciate your comment and consider it as a recommendation for a secondary analysis. |
| Discussion:  papers (28)…one study, where? | Thank you. Added. |
| “Pharmacists in developed countries are considered approved and reliable vaccination providers which boosts vaccination coverage and healthcare financial savings (31, 32, 33). Therefore, it is important to promote pharmacists’ role as vaccination providers to spread awareness and a more positive attitude towards vaccination-related advantages. This is not included in your research purpose. | Thank you. Added! |
| Discussion needs to focus on the results as well as the applicability of the study. | The recommendations section is updated. |
| Over all review:  English, well written. The study needs further analysis to assess the related factors, clearness regarding the elective vaccination was not found in the study making the study results very predictable.  Thank you, | Thank you for your recommendations. The current study is exploratory in nature, we will consider your comments in secondary analyses and/or studies. |
